# Contrasting diel hysteresis between soil autotrophic and heterotrophic respiration in a desert ecosystem under different rainfall scenarios

**DOI:** 10.1038/srep16779

**Published:** 2015-11-30

**Authors:** Weimin Song, Shiping Chen, Yadan Zhou, Bo Wu, Yajuan Zhu, Qi Lu, Guanghui Lin

**Affiliations:** 1 Ministry of Education Key Laboratory for Earth System Modeling, Center for Earth System Science, Tsinghua University, Beijing 100084, China; 2 State Key Laboratory of Vegetation and Environmental Change, Institute of Botany, Chinese Academy of Sciences, Beijing 100093, China; 3 Institute of Desertification Studies, Chinese Academy of Forestry, Beijing 100091, China

## Abstract

Diel hysteresis occurs often between soil CO_2_ efflux (R_S_) and temperature, yet, little is known if diel hysteresis occurs in the two components of R_S_, i.e., autotrophic respiration (R_A_) and heterotrophic respiration (R_H_), and how diel hysteresis will respond to future rainfall change. We conducted a field experiment in a desert ecosystem in northern China simulating five different scenarios of future rain regimes. Diel variations of soil CO_2_ efflux and soil temperature were measured on Day 6 and Day 16 following the rain addition treatments each month during the growing season. We found contrasting responses in the diel hysteresis of R_A_ and R_H_ to soil temperature, with a clockwise hysteresis loop for R_H_ but a counter-clockwise hysteresis loop for R_A_. Rain addition significantly increased the magnitude of diel hysteresis for both R_H_ and R_A_ on Day 6, but had no influence on either on Day 16 when soil moisture was much lower. These findings underline the different roles of biological (i.e. plant and microbial activities) and physical-chemical (e.g. heat transport and inorganic CO_2_ exchange) processes in regulating the diel hysteresis of R_A_ and R_H_, which should be considered when estimating soil CO_2_ efflux in desert regions under future rainfall regime.

Soil CO_2_ efflux, the second largest terrestrial carbon flux following photosynthesis, has received widespread attention during the past decades due to its vulnerability and high sensitivity to climate change^1^,^2^,^3^. On a diel, seasonal and annual scale, soil temperature is widely recognized as a primary abiotic factor affecting soil CO_2_ efflux, and the relationship between soil CO_2_ efflux and soil temperature is often expressed as Arrhenius or van’t Hoff exponential functions^4^,^5^,^6^. However, describing and predicting soil CO_2_ efflux using these simple reaction functions lead to many uncertainties, because various biotic and abiotic factors, such as plant physiological activity, soil physical properties, and soil moisture^4^,^7^,^8^,^9^, can also considerably influence the relationship.

In recent years, many field studies have shown that, on the diel and seasonal scales, soil CO_2_ efflux and soil temperature are decoupled, showing an elliptical hysteresis loop^10^,^11^. For example, soil CO_2_ efflux may increase more quickly in response to rising soil temperature in the morning compared to decreasing soil temperature in the evening, resulting in a clockwise diel hysteresis. Diel hysteresis (in some references also as “diurnal hysteresis”) has been observed in the soils of forests^12^,^13^,^14^,^15^, savanna grasslands^16^,^17^, and crop fields^18^,^19^,^20^. The mechanistic explanation for such hysteresis phenomenon of soil CO_2_ efflux is still lacking, although two possible hypotheses have emerged. The first one focuses on the biological mechanism associated with photosynthate transport such that recent photosynthates are periodically transported to the roots zone, where they can alter the magnitude and timing of soil CO_2_ efflux, leading to the phenomenon that soil CO_2_ efflux is out of sync with soil temperature^13^,^15^,^21^. For example, by comparing the relationship between soil CO_2_ efflux and soil temperature under tree canopies and in an open area, Vargas and Allen^13^ proposed that photosynthesis is the main contributor to the diel hysteresis phenomenon of soil CO_2_ efflux. The second hypothesis relies on the physical mechanisms associated with heat transport and CO_2_ diffusion processes. The direction of heat transport is opposite in daytime to that in nighttime, which can influence the velocities of CO_2_ diffusion along soil profiles when soil warms and cools^10^,^15^. The biological and physical mechanisms are not mutually exclusive, and both are likely to play important roles in formulating diel hysteresis of soil CO_2_ efflux^10^. In addition, many researchers have suggested that without considering diel hysteresis, a large bias will likely be present in the estimation of soil CO_2_ efflux and soil C loss^15^,^17^,^22^. In a montane conifer forest, for example, Riveros-Iregui *et al.*^15^ found that a representative model without considering diel hysteresis could overestimate the total soil CO_2_ efflux of the growing season by 19%. Thus, proper interpretation and evaluation of diel hysteresis of soil CO_2_ efflux is critical to accurate estimation and prediction of regional and global C budgets.

Soil rewetting following a rainfall event has been shown to be an important controlling factor for regulating soil CO_2_ efflux, especially in desert and semiarid regions^8^,^23^,^24^. Global climate models predict that the desert and semiarid regions such as those in northern China will undergo more extreme climate changes characterized by increasing amount of total precipitation and higher frequency of extreme rainfall events in the 21st century^25^,^26^,^27^. Given that water is the primary driver of biological activity in desert and semiarid areas^28^, such alterations of the hydrological cycle will potentially alter soil CO_2_ efflux. In addition, by changing soil moisture, precipitation increase can influence heat transport and gas diffusion processes, since diffusion of gas in the gas phase is much higher than in the liquid phase^29^. Therefore, any change in precipitation regions under the climate change scenarios can potentially influence diel hysteresis of soil CO_2_ efflux. Thus, it is very important to evaluate possible responses of diel hysteresis of soil CO_2_ efflux to changes in soil water availability associated with rainfall events, as any change in diel hysteresis of soil CO_2_ efflux may affect the evaluation of possible response of soil CO_2_ efflux to future precipitation change.

Soil CO_2_ efflux has two components: the autotrophic respiration (R_A_) of roots and the associated rhizosphere community, and the heterotrophic respiration (R_H_) from the decomposition processes of soil microbes^9^,^30^. However, most previous field studies mainly focused on diel hysteresis between total soil CO_2_ efflux and soil temperature^12^,^13^,^14^,^15^,^16^,^17^,^19^. Research concerning the diel hysteresis of the two respiration components is still rare^18^ and represents a gap in our knowledge of the response of soil CO_2_ efflux to temperature. In addition, we are not aware of any study conducted to distinguish the differential response of diel hysteresis between the two components of soil CO_2_ efflux *in situ* during rain pulses over an entire growing season. Modeling studies have shown differential responses of R_A_ and R_H_ to climate change such as increasing temperature^31^, indicating that the responses would be accompanied by varying changes in the magnitude of diel hysteresis of the two respiration components. Thus, partitioning soil CO_2_ efflux into its two components, and analyzing the different responses of diel hysteresis are urgently needed for more accurate modeling of regional and global C budgets under future climate change scenarios.

We conducted a manipulative field experiment to simulate five scenarios of future rain regimes (0%, 25%, 50%, 75% and 100% increase over local annual mean precipitation (115 mm)) in a desert ecosystem of northwestern China dominated by the shrub species *Nitraria tangutorum*. The rain addition treatments were applied equally each month during the growing season (May-September). The effects of rain addition amount and timing on soil CO_2_ efflux in this arid ecosystem have been reported previously^8^,^32^. Although this study used some of the same dataset as our previous study^32^, the current study focused on the diel hysteresis phenomenon of two soil respiration components and their differential responses to rain additions, which is quite different from our previous paper in term of the scientific questions we addressed and the methods we used to analyze the data. The main objectives of this paper were to evaluate: (1) the diel relationship of the two components of soil CO_2_ efflux with soil temperature, and (2) the possible responses of the diel hysteresis of the two components to rain increase. We hypothesized that the responses of diel hysteresis to rain addition would differ between the two components of soil CO_2_ efflux because roots and microbes often have different response mechanisms to rain pulses of varying size, considering that small rainfall events can only enhance microbial respiration and larger rainfall events can increase both microbial and root respiration with different response time and duration^24^,^32^,^33^.

## Materials and Methods

### Study site description

The experimental site (38° 34′ N, 102° 58′ E) was located in a sandy land area between Badain Jaran Desert and Tengger Desert in Minqin County, Gansu province, China. This area has a temperate arid continental climate with a mean annual temperature of 7.8 °C. The temperature during the warmest and coldest months is 23.2 °C and −9.6 °C, respectively. Mean annual precipitation is 115 mm mainly from July to September. The dominant soil type is aeolian sandy soil (Entisols in the USDA soil taxonomy system) with pH about 8.6. The dominant species is *Nitraria tangutorum Bobr*. More detailed information on the physical and chemical properties of soil can be found in Song *et al.*^8^.

### Experimental design

The experiment was carried out in a patchy landscape with *N. tangutorum* interspersed with sand dunes, oriented in a northwest-southeast direction and composed of two distinct types of soil cover. The northwest area was covered with *N. tangutorum* plants, while the southeast area was bare soil. The mean height of the dunes was 0.9 m and the area was 14.2 m^2^. Vegetation cover was approximately 35% at the entire study site.

A completely random design was used in this experiment, with five precipitation treatments and four replicates for each treatment (113 m^2^ per plot, 20 plots in total). During the growing season (from May to September) of 2009, five precipitation treatments were designed to simulate a rain increase of 0% (CK), 25% (+25%PPT), 50% (+50%PPT), 75% (+75%PPT) and 100% (+100%PPT) over long-term average annual precipitation at the study site (115 mm). The precipitation addition treatments were applied every month and the precipitation amount added each time was 0, 5.8, 11.5, 17.3 and 23.0 mm for the five addition treatments, respectively. Water was pumped into a tank from a well near the plots and used to irrigate the plots via an irrigation system composed of a water-pump, water meter and spraying arms. In order to reduce water evaporation, precipitation treatments were carried out only in the morning. More detailed information on this experiment is provided in Song *et al.*^8^.

### Soil CO_2_ efflux measurements

Soil CO_2_ efflux was measured on Day 6 and Day 16 after each rain addition for each month during the growing season (May to September). According to the general model of ecosystem carbon exchange following rain pulses^24^,^33^,^34^, shrubs in desert ecosystems are generally characterized by long lag time and long duration relative to microbes in responding to an effective rainfall. Huang and Nobel^35^ found that desert plants initialized new root growth c. 7 days after rewetting. The rate of soil CO_2_ efflux of vegetated soils in water irrigated plots showed a significant difference from that in control plot only about 1 week after the water addition treatment^8^. In addition, the soil moisture data also showed that the effects of the rain addition treatments could last as long as 15 days after the rain addition treatment^8^. Thus, we chose Days 6 and 16 after the rain addition treatment for the soil CO_2_ efflux measurements in this study.

At the beginning of the experiment, two PVC soil collars (80 cm^2^ in area and 5 cm in height) were installed into the vegetated and bare soils to a depth of 3 cm in each plot for measuring soil CO_2_ efflux. The rate of soil CO_2_ efflux was determined using an automated soil CO_2_ flux system (LI-8100, LI-COR Inc., Lincoln, NE, USA). Soil CO_2_ efflux was measured on Days 6 and 16 after rain addition each month during the growing season (May to September). On each measurement day, soil CO_2_ efflux rate was measured on each pre-installed collar repeatedly at an interval of 3 h from 6:00 AM to 6:00 AM of the following day. To achieve a more accurate estimate of soil CO_2_ efflux, we visited the 20 plots in the same sequence on every occasion that measurements were taken. As the rate of soil CO_2_ efflux was being measured, soil temperature (Ts) was measured at a depth of 10 cm with a thermocouple connected to the LI-8100 system. On each given measurement day, we also measured daily soil gravimetric water content (SWC) of the top10 cm of the soil column in all plots by the oven drying method. We chose to measure soil temperature at 10 cm depth based on the compromise that most microbes were concentrated in the upper layers (0–10 cm). However, most roots biomass was found in the 10–30 cm soil layers.

### Partition of soil CO_2_ efflux components

At the study site, no obvious root biomass existed in the bare soils, and grasses and forbs were scarce enough to be negligible. Thus, the soil CO_2_ efflux in the bare soils would mainly come from the decomposition of soil organic carbon - microbial respiration or heterotrophic respiration (R_H_). There were no differences in soil organic matter, soil nitrogen content or microbial biomass between the two vegetation cover types^8^. In addition, there were no differences in soil temperature or moisture between the two vegetation cover types during the experimental period. Thus, autotrophic respiration (R_A_) could roughly be determined by the difference in soil CO_2_ efflux measured in the vegetated and bare soils:


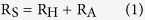


where R_S_ is soil CO_2_ efflux in the vegetated soil; R_H_ is soil CO_2_ efflux in the bare soil; and R_A_ is autotrophic respiration.

### Quantification of the diel hysteresis for soil CO_2_ efflux

For each measurement day of the growing season, we fitted the relationship between soil CO_2_ efflux and soil temperature during the night time (21:00–06:00) using a van’t Hoff exponential function. The parameters of the function were then adopted to calculate soil CO_2_ efflux during the daytime (09:00–18:00). Diel hysteresis was determined by the maximum difference of the daytime soil CO_2_ efflux measured and calculated.


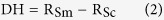


where the degree of diel hysteresis (DH) is the maximum difference between the soil CO_2_ efflux measured (R_Sm_) during the daytime and the efflux calculated by the exponential function (R_Sc)_ that was extracted from the relationship between the nighttime soil CO_2_ efflux and soil temperature. In this study, the maximum difference occurred at 12:00 for each measurement day. Positive values of diel hysteresis represent clockwise loop (higher CO_2_ efflux during soil warming period).

### Statistical analyses

We performed the repeated measurement analysis of variance (ANOVA) to test the differences in the degree of diel hysteresis of R_S_, R_H_ and R_A_ on Days 6 and 16 after different rain addition treatments. Two-way ANOVA tests were used to examine the effect of rain addition time and rain addition amount on diel hysteresis of R_S_, R_H_ and R_A_ on Days 6 and 16 after rain addition. Linear regression analysis was conducted to examine possible relationships between diel hysteresis and daily soil moisture. All statistical analyses were performed using SPSS 17.0 (SPSS for Windows, Version 17.0, Chicago, IL, USA).

## Results

### Soil water content and temperature under different rain additions

**Figure 1:**
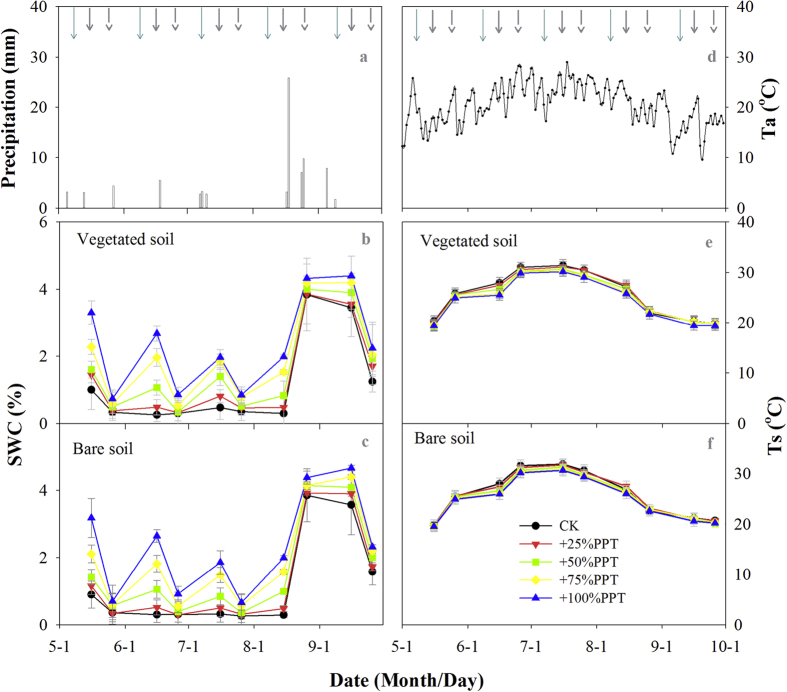
(**a**) Daily precipitation during the experimental period from 1 May to 1 October. (**b**,**c**): Soil water content (SWC) at 10 cm depth in vegetated soil and bare soil of a *N tangutorum* dominated desert ecosystem. (**d**) Daily mean air temperature (Ta) during the experimental period. (**e,f**): Soil temperature at 10 cm depth (Ts) in vegetated and bare soils. Long solid arrows represent the timing of rain addition treatments; short solid and dashed arrows represent the measurement time on Day 6 and Day 16 after the rain addition treatments, respectively.

We measured the surface (0–10 cm) soil water content (SWC) on Days 6 and 16 following the rain addition treatments each month. As expected, SWC was strongly affected by rain addition treatments in both vegetated and bare soils (Fig. 1). The magnitude of change in SWC depended on the amount of rain added and the measurement day. Day 6 usually showed a higher increase in SWC than Day 16 during the growing season except for August due to a heavy natural rainfall event on 25^th^August (25.8 mm). There was no significant difference in SWC between the vegetated and bare soils under a given rain addition treatment on each measurement day^32^.

The seasonal trend in daily mean surface (0–10 cm) soil temperature (Ts) was similar for both vegetation cover types during the entire growing season (Fig. 1). Daily mean Ts at each measurement day did not show any significant differences among the five rain addition treatments^32^. Moreover, there was no difference in daily mean Ts between the vegetated and bare soils under a given rain addition treatment on each measurement day^32^.

### Relationship between diel soil CO_2_ efflux and soil temperature

**Figure 2:**
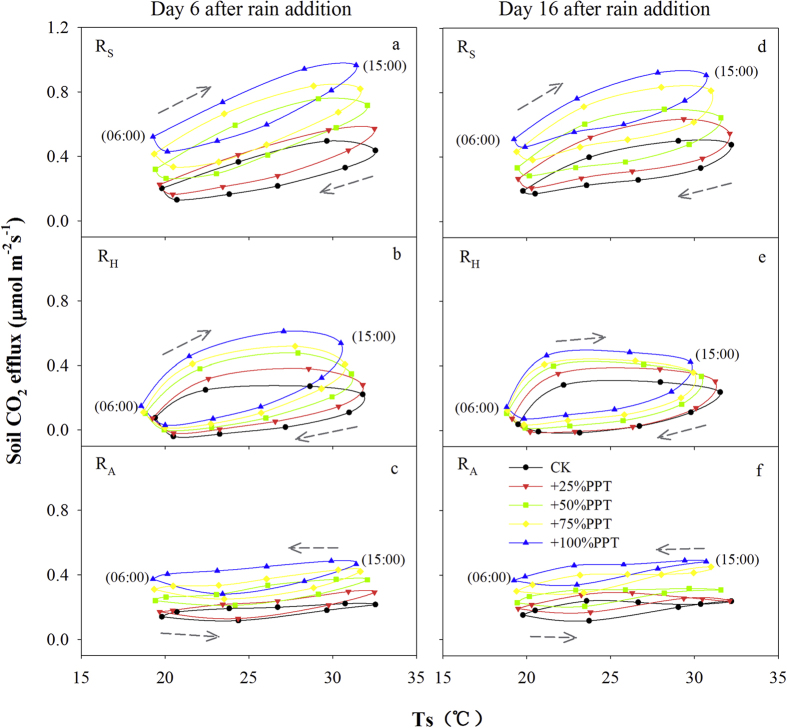
Relationships between diel soil CO_2_ efflux and soil temperature at 10 cm soil depth on Days 6 and 16 after the rain addition treatments during the growing season. (**a,d)** Soil CO_2_ efflux in the vegetated soils (total soil CO_2_ efflux, R_S_), (**b,e**) soil CO_2_ efflux in the bare soils (heterotrophic respiration, R_H_), (**c,f**) calculated autotrophic respiration (R_A_). Dotted arrows indicate the directions of the hysteresis loop.

Plots of the diel dynamics of R_S_, R_H_ and R_A_ against soil temperature on Day 6 and Day 16 following the rain addition treatments are shown in Fig. 2. A clockwise diel pattern of hysteresis with respect to soil temperature was found for R_S_ and R_H_ on both measurement days, with higher rate of R_S_ and R_H_ when soil temperature was increasing during daytime, and lower rate when temperature was decreasing during nighttime (Fig. 2a,b,d,e). The rate of R_S_ and R_H_ reached the maximum value between 12:00–15:00 and then gradually decreased to minimum value between 3:00–6:00. However, the maximum rate of R_A_ occurred between 18:00–21:00, and the minimum occurred between 6:00–9:00 despite the increase in soil temperature (Fig. 2c,f). Diel rate of R_A_ exhibited a counter-clockwise effect with soil temperature, that is, for a given temperature, the rate of R_A_ at nighttime was higher than at daytime.

### Effect of rain addition treatments on diel hysteresis of soil CO_2_ efflux

**Figure 3:**
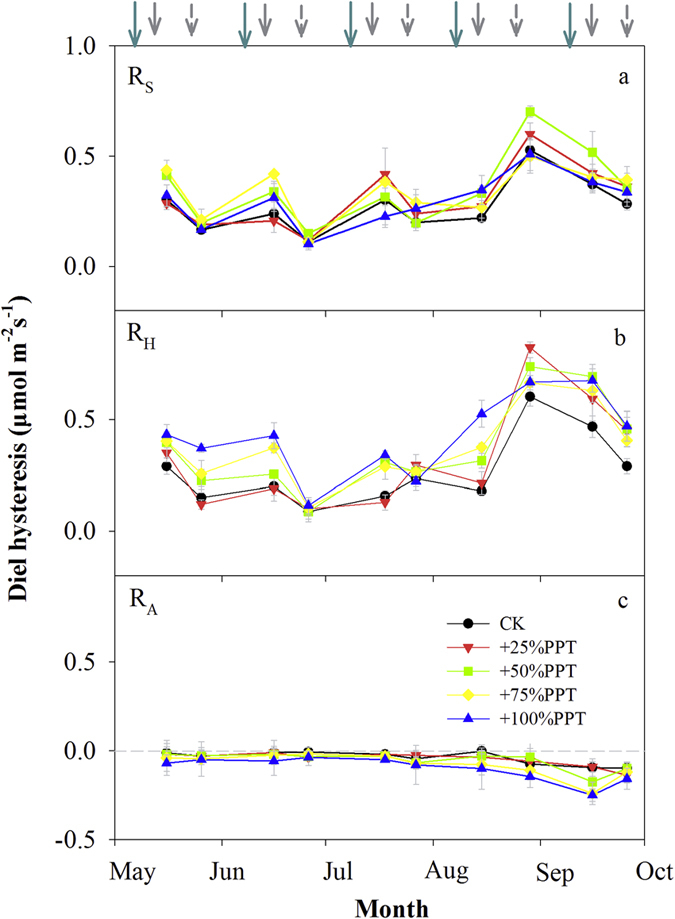
Seasonal variations in the diel hysteresis of soil CO_2_ efflux (R_S_) and its components (R_H_ and R_A_). Long solid arrows represent the timing of rain addition treatments; short solid and dashed arrows represent the measurement time on Day 6 and Day 16 after the rain addition treatments, respectively.

During the growing season, the diel hysteresis of R_S_ and R_H_ was positive while the diel hysteresis of R_A_ was negative (Fig. 3). The values of diel hysteresis of R_S_ and R_H_ showed significant seasonal variations, with the minimum diel hysteresis of R_S_ and R_H_ occurring on June 26^th^ and the maximum on August 26^th^ (Fig. 3a,b). However, the diel hysteresis of R_A_ were relatively stable during the growing season (Fig. 3c).

**Figure 4:**
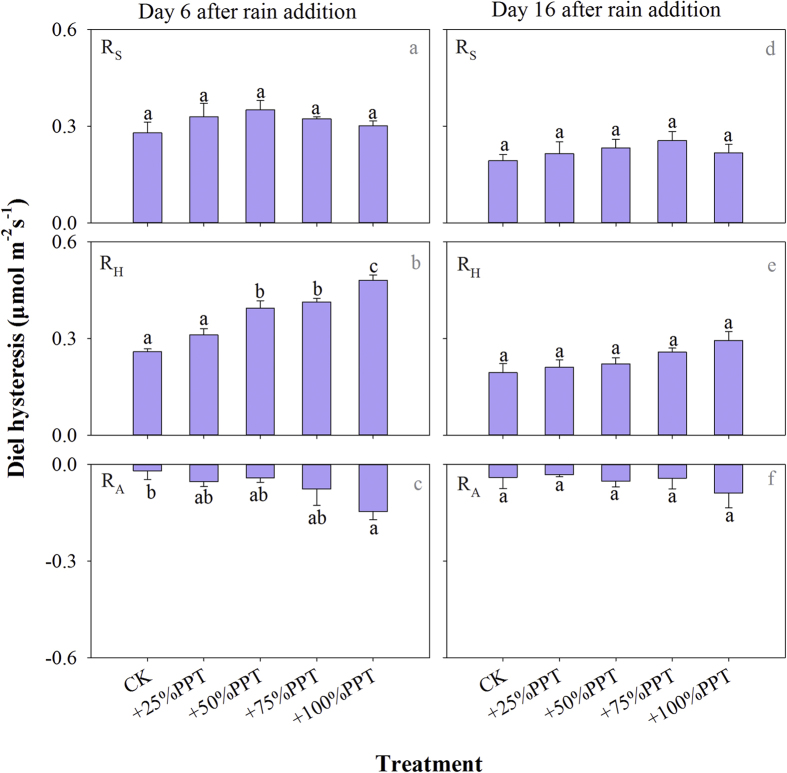
Mean values of diel hysteresis of soil CO_2_ efflux (R_S_) and its components (R_H_ and R_A_) on Day 6 and Day 16 after different rain addition treatments during the growing season in 2009 (n = 4). The different letters indicate significant differences between rain addition treatments at *P* < 0.05.

**Figure 5:**
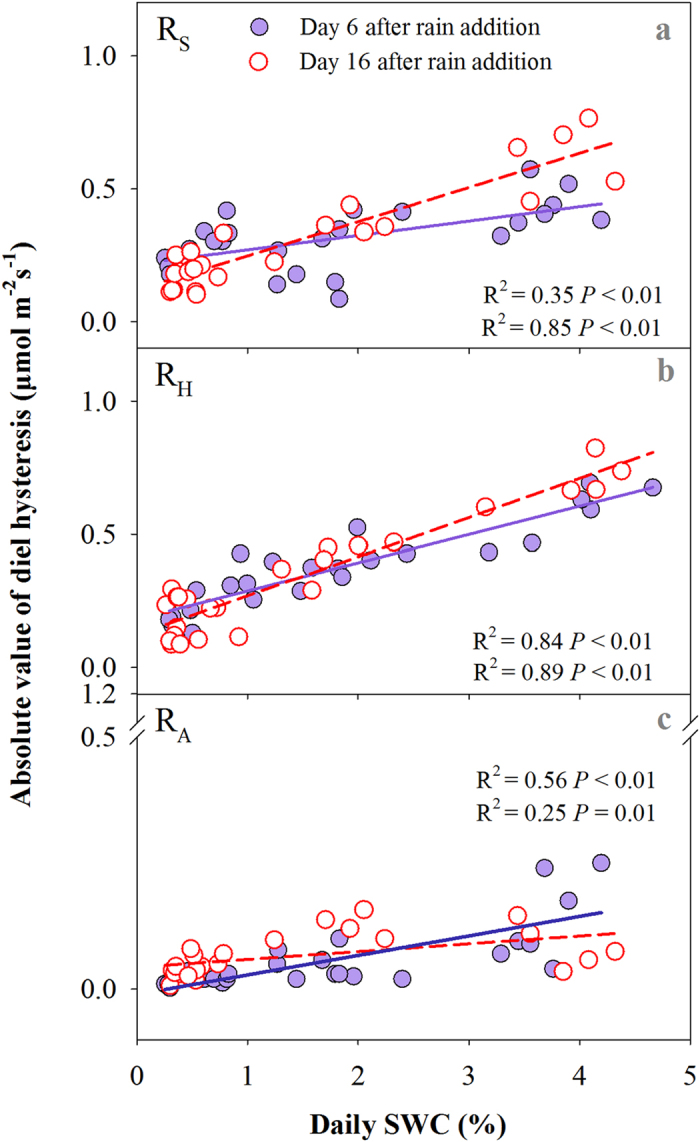
Relationships between absolute values of diel hysteresis and daily soil water content (SWC).

On Day 6, the rain addition had no influence on the diel hysteresis of R_S_ (Fig. 4a), but significantly increased the amplitudes of diel hysteresis of the two respiration components (Fig. 4b,c). On Day 16, the rain addition had no significant effect on the diel hysteresis of R_S_ and its components (Fig. 4d-f). There was no significant interaction effect of rain addition amount and rain addition time on the diel hysteresis of R_A_ and R_H_ on either Day 6 or 16 (Table S1). The value of diel hysteresis for R_H_ was always higher than for R_A_ on both measurement days. In addition, the amplitude of diel hysteresis positively corresponded with he increase in daily SWC for R_S_ and its components on both measurement days (Fig. 5).

The method of using nighttime soil CO_2_ efflux data to predict daytime soil CO_2_ efflux, a common method in eddy covariance studies, resulted in significant error in the estimation of daily total soil CO_2_ efflux (Table 1). During the growing season, there was an underestimation of daily flux by 10–32% for R_S_ and 41–62% for R_H_, but an overestimation of R_A_ by 5–12% in this desert ecosystem. Rain addition changed the value of the error, and the magnitude of the change depended on the date of measurement (Table 1). On Day 6, rain addition significantly decreased the magnitude of the error for R_S_ and R_H_ but had no influence for R_A_. On Day 16, however, there was a significant decrease in the magnitude of error for R_S_ but had no change in the two respiration components.

## Discussion

Numerous studies have investigated diel hysteresis between soil CO_2_ efflux and temperature, but reached contrasting conclusions on the mechanisms for the occurrence of such phenomenon^12^,^13^,^14^,^15^,^16^,^17^,^18^,^19^,^20^,^36^. In addition, these results mainly focused on diel hysteresis of total soil CO_2_ efflux and so far research concerning the diel hysteresis of two respiration components is still rare^18^. Furthermore, how diel hysteresis will respond to climate change, particularly precipitation change, is still poorly understood. To best of our knowledge, this is the first study focused on the differential responses of the diel hysteresis between the two components of soil CO_2_ efflux *in situ* to rain pulses over an entire growing season. Our results from this field manipulation experiment offer new insights into how soil CO_2_ efflux responds to temperature and soil moisture.

In this study, we found that a distinct diel hysteresis existed between R_A_ and R_H_ in terms of their responses to soil temperature. The rate of R_A_ was higher when soil temperature was decreasing than when soil temperature was increasing, producing a counter-clockwise hysteresis loop. In contrast, the rate of R_H_ increased more quickly in response to rising soil temperature in the morning compared to decreasing soil temperature in the evening, showing a clockwise hysteresis loop. These results suggest that both biological and physical mechanisms should be responsible for the diel hysteresis of soil CO_2_ efflux in this desert ecosystem, and also indicate that variables that control diel behaviour of R_A_ in response to changes in soil temperature are different from the variables that control diel behaviour of R_H._

**Figure 6:**
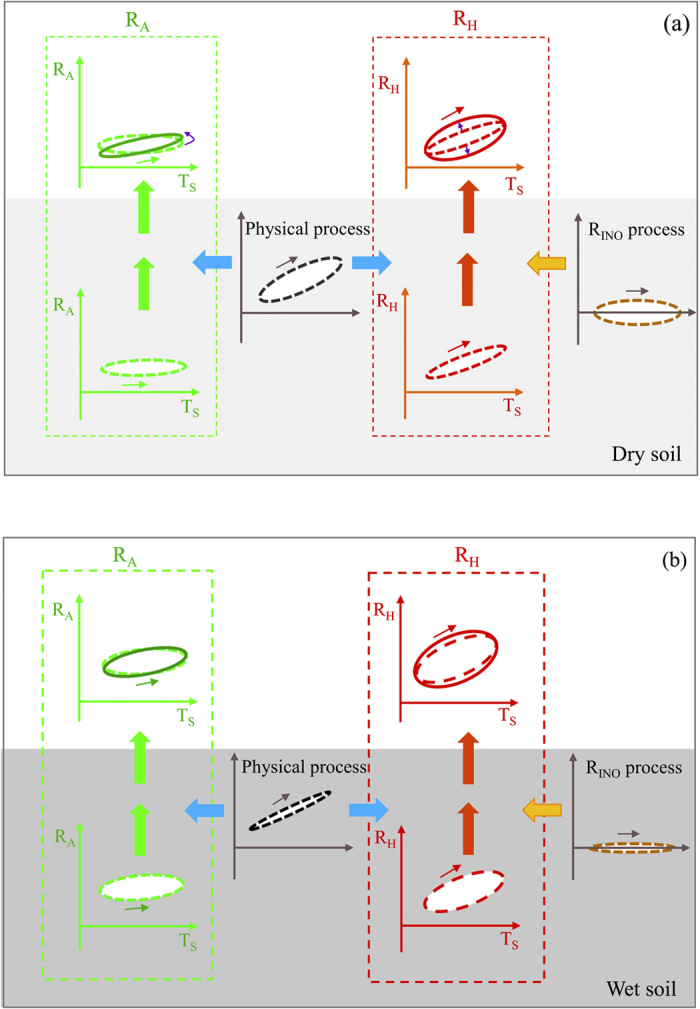
Conceptual illustration of factors controlling diel hysteresis patterns of soil CO_2_ efflux (R_S_) and its components (R_A_ and R_H_) in responding to soil temperature in dry (a) and wet (b) soils. The effect of physical (mainly heat) transport processes on the diel hysteresis of R_A_ and R_H_ is same. Besides physical process, the diel hysteresis of R_H_ is also modified by chemical (inorganic CO_2_ efflux, R_INO_) process. In dry soil condition (**a**), the effects of physical and chemical processes to the diel relationship between soil CO_2_ efflux and soil temperature are pronounced, while the effects are limited under wet soil condition (**b**). See the text in the Discussion for more details.

Several field studies have found a strong connection between plant photosynthesis and R_A_ at diel and seasonal scales^16^,^21^,^37^. The time required to transport photosynthetic C to roots was found on the order of many hours to a few day^10^,^13^,^15^,^38^. For instance, Tang *et al.*^16^ reported that the time for photosynthetic products to be transported to the roots and be respired is less than 1 day. Similarly, by *in situ*^13^CO_2_ pulse labelling of shrubs, Carbone and Trunbore^39^ demonstrated that a part of photosynthetic C can be rapidly transported to belowground and be quickly metabolized by roots within a few hours. In this study, peak R_A_ occurred at between 18:00–21:00, which was about 3 h after peak 10 cm soil temperature, 6 h after peak PAR. The high correlation between daily mean PAR and daily mean R_A_ (Fig. S1) suggested that the C respired by roots may be related to the same day’s photosynthesis. We speculate that accumulated photosynthetic C during daytime was periodically transported to the roots and metabolized at night, which could have contributed to the counter-clockwise diel hysteresis of R_A_ (Fig. 6a). However, in this study site, most roots biomass was concentrated in the 10–30 cm soil layers, which suggested that the diel hysteresis of R_A_ could have also been modified by physical processes. According to model analysis, Phillips *et al.*^10^ demonstrated that the effect of CO_2_ diffusivity on diel hysteresis can be bypassed because it does little to shift diel oscillation of R_A_ considering the high porosity of sandy soil, which is similar to the soil type in this study. In contrast to CO_2_ diffusivity, model based on physical first principles (Table 1 and Fig. 3 in Phillips *et al.*^10^) proved that thermal transport plays an important role in controlling the diel hysteresis of soil CO_2_ efflux by controlling the speed of air temperature propagated through soil and by changing the synchronicity of diel variations of soil temperature and efflux^10^. The asynchronous diel patterns between soil temperature and efflux could result in a clockwise hysteresis loop within an approximate rate (e.g. 1 × 10^−7^ m^2^ s^−1^ based on measurements of a sandy loam soil) of thermal diffusivity for sandy soils experiencing normal field moisture level^10^ (Fig. 6a). Thus, although the clockwise effect of physical processes had no influence on the rate of R_A_ and the rotational direction of hysteresis loop, it could have modified the orientation of the principal axes of the hysteresis loop of R_A_ (Fig. 6a). Consequently, the co-occurrence of biological mechanism (diel photosynthetic carbon supply) and physical processes, particularly thermal transport, could have contributed to the counter-clockwise diel hysteresis of R_A_ in this desert ecosystem.

On the other hand, the clockwise hysteresis effect of R_H_ could not be associated with carbon supply mechanism because there were no obvious root biomass in the bare soils. The explanation for the diel hysteresis of R_H_ is still unclear, but three possible mechanisms may account for it. First, physical processes associated with heat transport and CO_2_ diffusion mentioned above may be responsible for the diel hysteresis of R_H_ (Fig. 6a). Second, many previous studies have reported that in arid ecosystems with saline/alkaline soils, there is a chemical (i.e. carbonate precipitation and dissolution) process (inorganic CO_2_ efflux, R_INO_) influencing the diel variation of soil CO_2_ efflux because R_INO_ shows a positive value during daytime (CO_2_ emission) and a negative value (CO_2_ absorption) at nighttime^40^,^41^,^42^,^43^ (Fig. 6a). Based on the diel relationship between R_INO_ process and soil temperate in Fig. 6a, which is redrawn based on results of Liu *et al.*^43^ and Ma *et al.*^40^ in saline/alkaline soil desert ecosystems located near our study site, we suggest that the diel variation in R_INO_ could have affected the observed diel hysteresis of R_H_ by modifying the rate of R_H_ and the roundness of hysteresis loop. However, the diel variation R_INO_ would have no influence on the orientation of the principal axes of the hysteresis loop of R_H_ and the rotational direction of hysteresis loop. Third, in desert ecosystems, microbial communities such as bacteria and fungi usually have different vertical distribution to compete for available substrates^44^,^45^,^46^. The differential temperature sensitivities of microbial components^47^,^48^,^49^ may have contributed to the diel hysteresis of R_H_. In addition, the growth rates of bacteria and fungi usually have optimum temperatures around 25–30 °C^49^. The higher temperature (above 33 °C) at noon at this site could have inhibited the activity of microbes, leading to a clockwise hysteresis loops. We propose that the above three mechanisms together resulted in the clockwise diel hysteresis of R_H_ (Fig. 6a). However, we found that R_H_ had a strong temporal coincidence with photosynthetic active radiation (PAR) (Fig. S2), which reached a maximum at 12:00. This is earlier than the soil temperature maximum, which occurred around 15:00, indicating that diel pattern of R_H_ in this desert ecosystem may be more closely related to near surface soil temperature. However, it does not necessarily imply that all microbial respiration occurs in surface soils, as microbial respiration at deep soils certainly occur.

In addition, a clockwise hysteresis loop of R_S_ was observed in responding to diel change of soil temperature, which is similar to that of R_H_ but distinct from that of R_A_. The clockwise diel pattern could be attributed to the high contribution of R_H_ to R_S_, particularly when soil temperature was increasing. Many previous studies suggest that the diel hysteresis between soil CO_2_ efflux and soil temperature is an artifact due to an arbitrary selection of a reference soil depth, which can produce an unrealistic relationship between efflux and soil temperature^19^,^50^. By exploring the relationship between soil CO_2_ efflux and its two components with air temperature (Fig. S3), we found a distinct, elliptical diel hysteresis, suggesting a potential occurrence of diel hysteresis of soil CO_2_ efflux in responding to soil temperature at different depths, and also highlighting the complexity of understanding the processes controlling the relationship between soil CO_2_ efflux and temperature. Thus, we argue that, in order to accurately understand the temperature dependence of R_S_, it is necessary to include measurements of soil temperature at several depths.

Rain addition resulted in a larger magnitude of diel hysteresis of R_A_ on Day 6 when soil moisture was higher. Many studies have shown that an effective rainfall can infiltrate into deep soils and be absorbed by plants with deep-rooted systems, leading to an increase in photosynthesis, especially in desert and semiarid ecosystems^24^,^51^. In this study, photosynthesis of *N. tangutorum* significantly increased after rain addition treatments (unpublished data). This higher photosynthesis of plants implied that a larger portion of photosynthetic C was transported into belowground and metabolized by roots^33^,^37^, which was of sufficient magnitude to alter diel patterns of R_A_^32^,^52^. However, increased soil water moisture following rain addition could have altered the effects of physical processes by increasing the rate of heat diffusivity and decreasing the rate of CO_2_ transport. The change in the two physical transport processes have consistent effect on diel hysteresis that cause clockwise diel hysteresis to become less pronounced^10^. Therefore, the limited effect of physical mechanism could have contributed to the change of diel hysteresis of R_A_ because physical processes had contrasting effect on the orientation of hysteresis loop. We found that rain addition had no effect on the diel hysteresis of R_A_ on Day 16 when soil was drier. The different effect of rain addition for the two days was most likely a consequence of variation in soil water content. On day 16, the lower soil water availability could have weakened the activity of plants and reduced the coupling between plant photosynthesis and belowground processes by reducing the movement of recent photosynthates to phloem loading sites and by changing the flow rate of photosynthates from the shoots into the roots^53^,^54^. This would potentially stifle the effects of rain addition on diel hysteresis of R_A_ on Day 16. In addition, the increasing effect of physical processes following soil drying could be responsible for the consistent values of diel hysteresis among treatments on Day 16.

Similarly, soil water availability is a vital factor in controlling microbial activity in desert and semiarid ecosystems^55^,^56^,^57^. After rewetting following simulated rain addition, microbial activity recovered quickly due to increased substrates availability and favorable environmental conditions^23^,^51^,^58^, leading to a large production of CO_2_ gas. In addition, the change in the two physical transport processes under rain addition treatments could have caused clockwise diel hysteresis to become less pronounced (Fig. 6b) because physical processes have a consistent effect on the orientation of hysteresis loop. Furthermore, increasing soil water content could decrease the effects of R_INO_ on diel hysteresis because the wetter the saline/alkaline soils are, the less effect R_INO_ will have on the diel pattern of R_H_^40^,^41^,^42^. Therefore, compared with dry soil (Fig. 6a) or control plots (CK), rain addition treatments could have diminished the effects of physical processes and R_INO_ on the diel hysteresis of R_H_.

Why was there an increase in the diel hysteresis of R_H_ on Day 6 after rain addition? We propose two possible mechanisms for this phenomenon. First, the high soil moisture enhanced the diffusion of organic molecules through soil water films and increased substrate availability for microbial mineralization^23^,^59^. This would have affected microbial activity differently in various soil layers because microbial communities have a vast vertical distribution and thus have different moisture sensitivities^47^,^48^,^49^, increasing the diel hysteresis of R_H_ on Day 6. Second, rain addition eliminated environment stress and provided a favorable (cool, wet) microclimate for microbial activity at a diel scale, particularly at noon^32^. The higher R_H_ rate and lower soil temperature at daytime resulted in an increase in the diel hysteresis of R_H_. Whereas on Day 16 when the soil was drier, the diel hysteresis of R_H_ decreased and showed no significant difference among treatments. Previous studies have shown that an effective rainfall event could trigger a rapid pulse response of microbial activity, and the response would gradually decrease due to exhaustion of accessible organic matter^55^,^60^,^61^. The declined substrate availability on Day 16 could have also inhibited the temperature response of microbial activity, particularly at midday when temperature reached maximum value, leading to a consistent diel hysteresis value of R_H_ among treatments. In addition, the consistent value could be attributed to the increasing role of physical mechanism discussed above. Our findings are consistent with other research showing that soil water availability can modify the magnitude of diel hysteresis between soil CO_2_ efflux and soil temperature^15^. In addition, we found that the change in soil moisture would only change the diel magnitude of R_H_ with no effect on orientation. These results derived from field experiment is consistent with theoretical analysis^10^.

Surprisingly, we found that rain addition had no influence on the diel hysteresis of R_S_ on the two measurement days, although the daily mean rate of R_S_ significantly increased by rain addition treatments on both days during the growing season^32^. In this study site, the increased size of clockwise hysteresis loop of R_H_ would be offset by the increased size of counter-clockwise hysteresis loop of R_A_, which could have resulted in the consistent value for diel hysteresis of R_S_ among the five rain addition treatments during the growing season.

In addition, we found that, without considering diel hysteresis, significant error would occur in the estimation of daily soil CO_2_ efflux based on a relationship between efflux and temperature. Thus, diel hysteresis should be considered in terrestrial ecosystem models for predicting regional C cycles in desert regions. The method of using nighttime soil CO_2_ efflux data to predict daytime soil CO_2_ efflux, a common method in eddy covariance studies, resulted in an underestimation of daily R_S_ and R_H_ by 10–32% and 41–62% respectively, but an overestimation of daily R_A_ by 5–12% in this desert ecosystem. However, in a Chihuahuan desert shrubland, Hamerlynck *et al.*^62^ showed an overestimation of daily soil CO_2_ efflux by 9–19% based on temperature-derived functions that do not consider diel hysteresis. Similarly, Riveros-Iregui *et al.*^15^ found an overestimation of growing season total soil CO_2_ efflux by 19% in a montane conifer forest when diel hysteresis was not considered. These results indicate that in different ecosystems with varying relative contribution of the two respiration components to the total efflux, the value of the error would be different. Thus, in order to accurately estimate ecosystem C budgets under future climate scenarios, we should consider the different responses of R_A_ and R_H_ to climate change, particularly to rainfall variability.

### Implications and limitations

The results from our field experimental studies have important implications for regional carbon cycle simulations. First of all, one can no longer confidently capture an entirety of temperature response in soil CO_2_ efflux by only measuring soil CO_2_ efflux late morning because of the present of hysteresis. At the ecosystem-scale, eddy covariance-based studies often use nighttime respiration data to predict daytime rates of respiratory efflux in accordance with exponential temperature extrapolation^63^. The results of this study underscore that the failure to account for possible hysteresis of soil CO_2_ efflux at diel and/or seasonal scales may result in under- or overestimation of ecosystem respiration^36^,^64^, which is composed of both soil and aboveground component efflux. In addition, the occurrence of diel hysteresis suggests a potential problem in the usage of constant temperature sensitivity of soil CO_2_ efflux for gap filling procedures and in ecosystem carbon cycling models^65^. Lastly, higher soil water content results in a larger amplitude of diel hysteresis, implying that the role of soil water content in controlling the relationship between soil CO_2_ efflux and soil temperature should be considered in the modeling of production and efflux of CO_2_ from the soils in desert and semiarid ecosystems subjected to severe seasonal changes in soil water availability.

Although we investigated the effects of experimental rain addition on diel hysteresis of soil CO_2_ efflux and its components (R_H_ and R_A_) in a desert ecosystem, this study was limited by several uncertainties. First, the calculation of diel hysteresis was based on the relationship between soil CO_2_ efflux and temperature at a soil depth of 10 cm. It is worth noting that the soil temperature used in our analysis is crucial because the phase and peak of soil temperature in different soil layers vary due to altered heat transport rate^19^,^50^, despite finding a distinct diel hysteresis between soil CO_2_ efflux and air temperature. Therefore, to understand exactly the diel hysteresis, several soil depth samples should be taken into account in further experiments. Second, the method of calculating R_A_ in our study was based on the difference in the rate of soil CO_2_ efflux between the vegetated soil and the bare soil; however, this method may ignore the presence of rhizosphere respiration. Thus our method may over- or under-estimate the diel response of R_A_ to soil temperature and result in a calculated deviation in diel hysteresis of R_A._ However, it should be noted that among the different partitioning methods reviewed by Hanson *et al.*^66^ and Kuzyakov^67^, our approach seems to be the most appropriate method for separating R_A_ and R_H_ in this desert ecosystem. Third, several previous studies suggested that a rainfall pulse after a period of drought can result in a quick response in microbial activity within several hours and the response is often short-lived in desert and semiarid regions^24^,^33^. In this study, we measured diel variations in soil CO_2_ efflux on Days 6 and 16 after the rain addition, which may not capture such quick response of microbial activity to the rain addition. Therefore, to fully understand the response of diel hysteresis to rainfall pulse, continuous soil CO_2_ efflux measurements following rainfall events should be performed in further experiments. Finally, this study was conducted in a desert ecosystem, and whether other ecosystems, such as grassland or forest ecosystems have a similar response in diel hysteresis of soil CO_2_ efflux and its components to rain addition is still unclear. Therefore, more experimental studies with better approaches (e.g. stable isotope labeling) are needed to better understand the diel hysteresis of soil CO_2_ efflux and its components as well as to accurately model soil C loss from terrestrial ecosystems under future climate scenarios.

## Additional Information

**How to cite this article**: Song, W. *et al.* Contrasting diel hysteresis between soil autotrophic and heterotrophic respiration in a desert ecosystem under different rainfall scenarios. *Sci. Rep.* **5**, 16779; doi: 10.1038/srep16779 (2015).

## Supplementary Material

Supplementary Information

## Figures and Tables

**Table 1:** A comparison of measured and calculated daily soil CO_2_ efflux (R_S_) and its components (R_H_ and R_A_) (g C m^−2^ d^−1^) that was based on the relationship between nighttime efflux and soil temperature on Days 6 and 16 after different rain addition treatments.

	R_S_			R_H_			R_A_		
	Measured	Calculated	Error	Measured	Calculated	Error	Measured	Calculated	Error
Day 6 after rain addition									
CK	0.31 (0.06)	0.21 (0.06)	−32 (3)^a^	0.11 (0.02)	0.04 (0.02)	−62 (2)^a^	0.20 (0.04)	0.21 (0.04)	6 (2)^a^
+25%PPT	0.44 (0.07)	0.31 (0.06)	−29 (2)^ab^	0.15 (0.03)	0.07 (0.03)	−56 (2)^ab^	0.19 (0.04)	0.21 (0.04)	10 (2)^a^
+50%PPT	0.51 (0.06)	0.40 (0.06)	−21 (2)^b^	0.20 (0.03)	0.09 (0.02)	−49 (3)^b^	0.31 (0.03)	0.32 (0.03)	4 (2)^a^
+75%PPT	0.67 (0.11)	0.59 (0.11)	−12 (3)^c^	0.22 (0.02)	0.11 (0.02)	−51 (1)^b^	0.45 (0.09)	0.47 (0.09)	5 (3)^a^
+100%PPT	0.73 (0.10)	0.66 (0.09)	−10 (3)^c^	0.27 (0.03)	0.13 (0.03)	−52 (2)^b^	0.47 (0.10)	0.50 (0.09)	6 (2)^a^
Day 16 after rain addition									
CK	0.33 (0.04)	0.26 (0.04)	−20 (2)^a^	0.13 (0.02)	0.07 (0.01)	−46 (2)^a^	0.20 (0.03)	0.23 (0.03)	15 (2)^a^
+25%PPT	0.40 (0.06)	0.31 (0.05)	−21 (3)^a^	0.15 (0.03)	0.08 (0.01)	−47 (2)^a^	0.25 (0.03)	0.27 (0.03)	8 (2)^a^
+50%PPT	0.48 (0.06)	0.39 (0.06)	−18 (2)^a^	0.20 (0.02)	0.11 (0.02)	−45 (1)^a^	0.28 (0.03)	0.31 (0.03)	11 (2)^a^
+75%PPT	0.58 (0.06)	0.49 (0.06)	−15 (2)^ab^	0.24 (0.02)	0.13 (0.02)	−44 (1)^a^	0.34 (0.05)	0.36 (0.05)	6 (3)^a^
+100%PPT	0.69 (0.06)	0.63 (0.06)	−12 (3)^b^	0.27 (0.01)	0.16 (0.01)	−41 (1 a	0.42 (0.05)	0.47 (0.05)	12 (3)^a^

Error = (Calculated-Measured) × 100%/Measured. A positive value of the Error represents an overestimation of the soil CO_2_ efflux, and *vice versa*. Values are presented as the mean (SE). The different letters within the Error column on each day indicate significant differences among the five rain addition treatments (*P *< 0.05).
