# Supplementary material for: Contrasting diel hysteresis between soil autotrophic and heterotrophic respiration in a desert ecosystem under different rainfall scenarios
Source: Sci Rep. 2015 Nov 30;5:16779. doi: 10.1038/srep16779 (PMC4663751; doi:10.1038/srep16779)
Supplement: Supplementary Information [file srep16779-s1.pdf]

Supplementary Information:

Contrasting diel hysteresis between soil autotrophic and heterotrophic respiration in a desert ecosystem under different rainfall scenarios

Weimin Song[a, b], Shiping Chen[b], Yadan Zhou[b], Bo Wu[c], Yajuan Zhu[c], Qi Lu[c] and Guanghui Lin[a*]

[a]Ministry of Education Key Laboratory for Earth System Modeling, Center for Earth System Science, Tsinghua University, Beijing 100084, China

[b]State Key Laboratory of Vegetation and Environmental Change, Institute of Botany, Chinese Academy of Sciences, Beijing 100093, China

[c]Institute of Desertification Studies, Chinese Academy of Forestry, Beijing 100091, China

*Corresponding author: Dr. Guanghui Lin, Email: lingh@tsinghua.edu.cn.

Supplementary Table S1. *P*-values of two-way ANOVAs on the effects of rain addition treatment (Treat) and rain time (Time) on diel hysteresis of soil CO_2 efflux (R_S) and its components (R_H and R_A) on Day 6 and Day 16 after rain addition.

	Day 6 after rain addition			Day 16 after rain addition		
	R_S	R_H	R_A	R_S	R_H	R_A
Treat	0.728	<0.001	0.001	0.348	0.058	0.094
Time	0.002	<0.001	0.460	<0.001	<0.001	0.654
Treat × Time	0.117	0.360	0.587	0.150	0.061	0.779

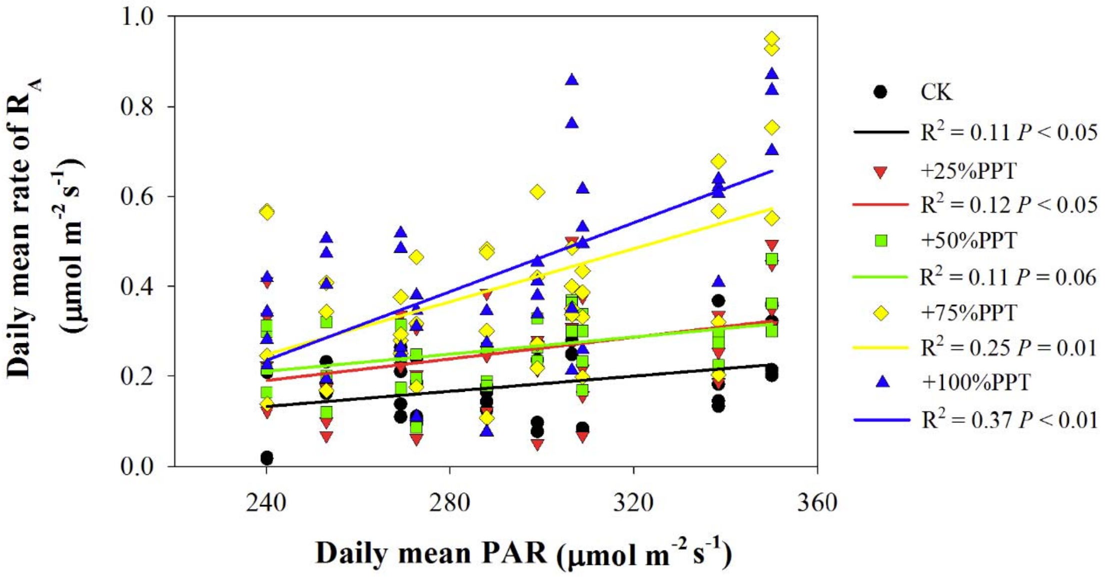

Supplementary Figure S1. Relationships between daily mean rate of autotrophic respiration (R_A) and daily mean photosynthetic active radiation (PAR) for the five rain addition treatments during the growing season.

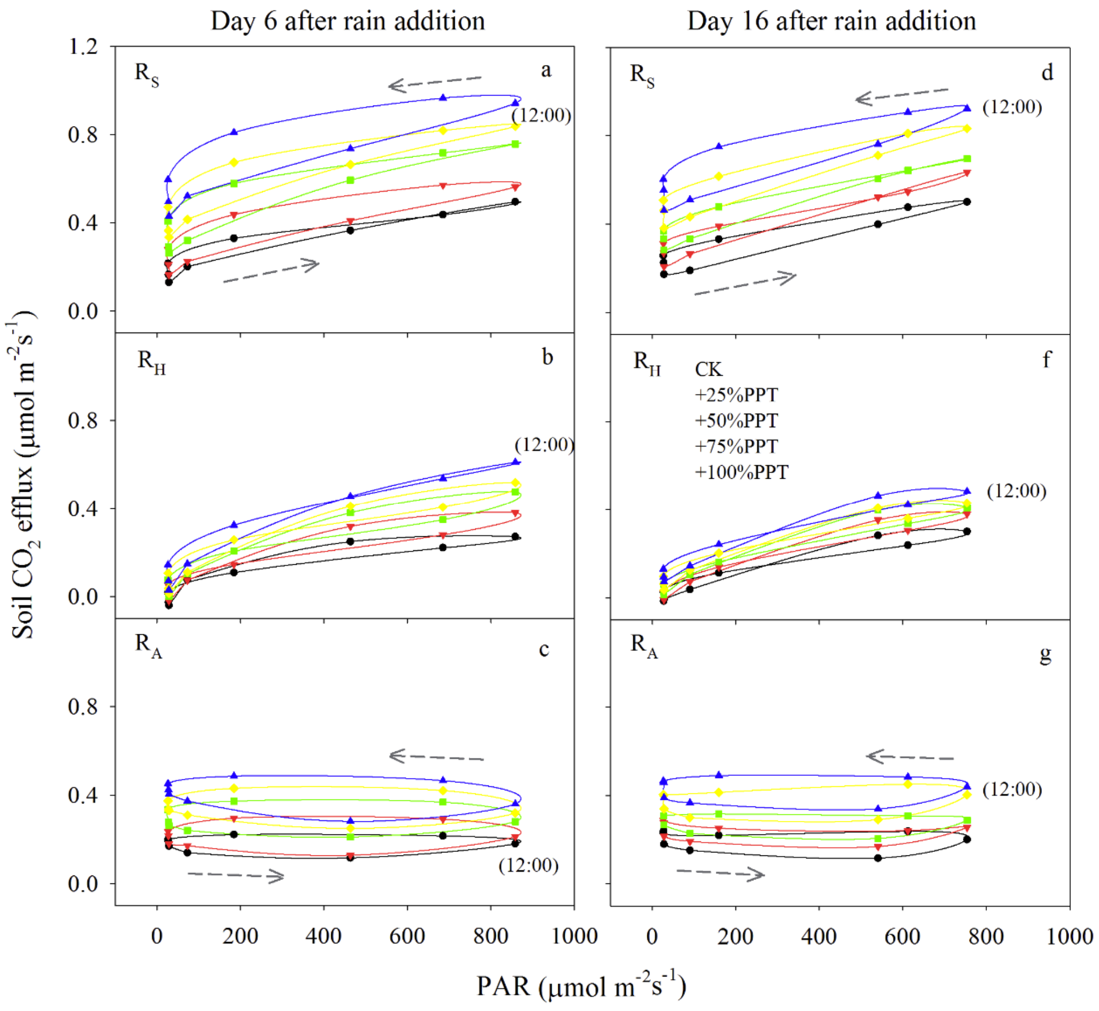

Supplementary Figure S2. Relationships between diel soil CO_2 efflux and its components with photosynthetic active radiation (PAR) on Day 6 and Day 16 after different rain addition treatments during the growing season. a and d: soil CO_2 efflux in the vegetated soils (total soil CO_2 efflux, R_S), b and e: soil CO_2 efflux in the bare soils (heterotrophic respiration, R_H), c and f: calculated autotrophic respiration (R_A).

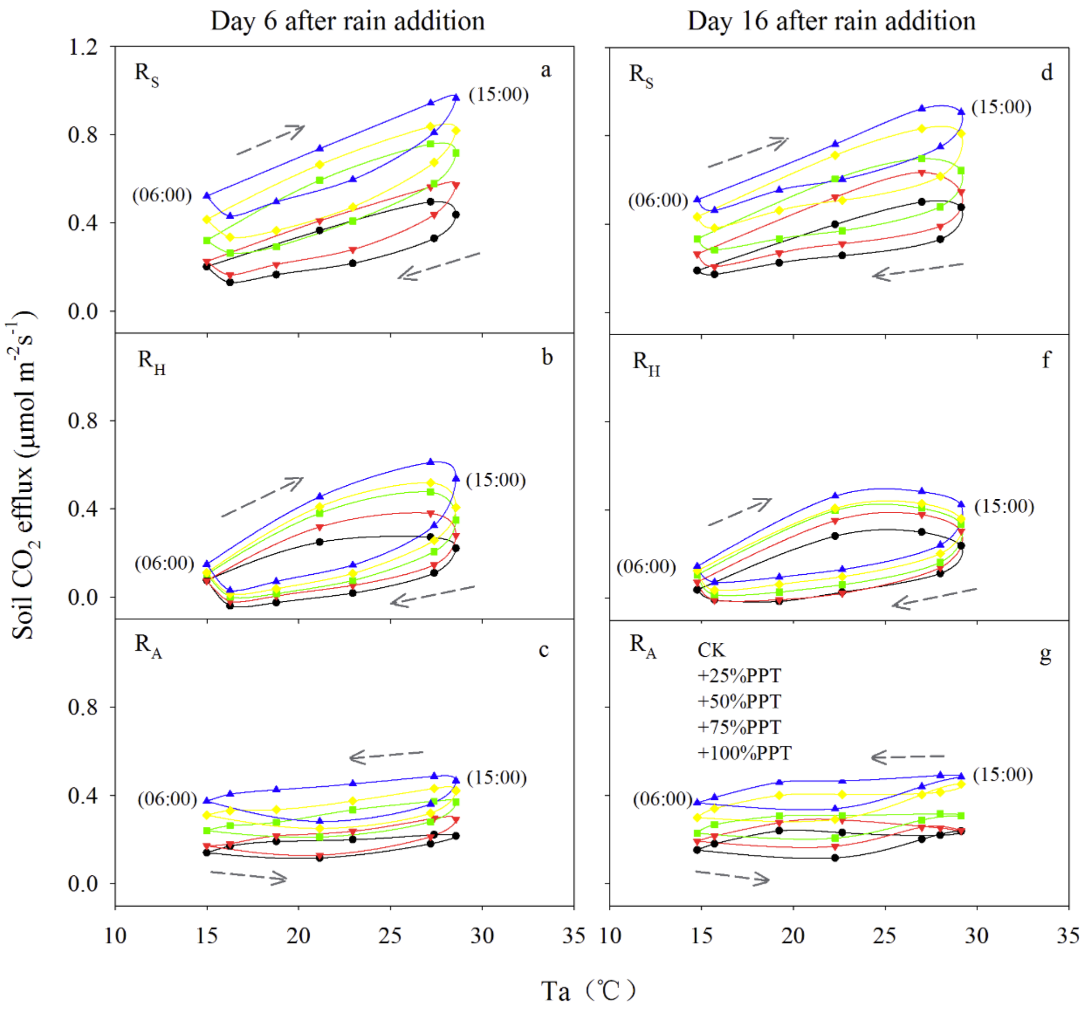

Supplementary Figure S3. Relationships between diel soil CO_2 efflux and its components with air temperature (Ta) on Day 6 and Day 16 after different rain addition treatments during the growing season. Dotted arrows indicate the directions of the hysteresis loop.
